# Finding type 2 diabetes causal single nucleotide polymorphism combinations and functional modules from genome-wide association data

**DOI:** 10.1186/1472-6947-13-S1-S3

**Published:** 2013-04-05

**Authors:** Chiyong Kang, Hyeji Yu, Gwan-Su Yi

**Affiliations:** 1 Department of Bio and Brain Engineering, KAIST, Daejeon 305-701, South Korea

## Abstract

**Background:**

Due to the low statistical power of individual markers from a genome-wide association study (GWAS), detecting causal single nucleotide polymorphisms (SNPs) for complex diseases is a challenge. SNP combinations are suggested to compensate for the low statistical power of individual markers, but SNP combinations from GWAS generate high computational complexity.

**Methods:**

We aim to detect type 2 diabetes (T2D) causal SNP combinations from a GWAS dataset with optimal filtration and to discover the biological meaning of the detected SNP combinations. Optimal filtration can enhance the statistical power of SNP combinations by comparing the error rates of SNP combinations from various Bonferroni thresholds and p-value range-based thresholds combined with linkage disequilibrium (LD) pruning. T2D causal SNP combinations are selected using random forests with variable selection from an optimal SNP dataset. T2D causal SNP combinations and genome-wide SNPs are mapped into functional modules using expanded gene set enrichment analysis (GSEA) considering pathway, transcription factor (TF)-target, miRNA-target, gene ontology, and protein complex functional modules. The prediction error rates are measured for SNP sets from functional module-based filtration that selects SNPs within functional modules from genome-wide SNPs based expanded GSEA.

**Results:**

A T2D causal SNP combination containing 101 SNPs from the Wellcome Trust Case Control Consortium (WTCCC) GWAS dataset are selected using optimal filtration criteria, with an error rate of 10.25%. Matching 101 SNPs with known T2D genes and functional modules reveals the relationships between T2D and SNP combinations. The prediction error rates of SNP sets from functional module-based filtration record no significance compared to the prediction error rates of randomly selected SNP sets and T2D causal SNP combinations from optimal filtration.

**Conclusions:**

We propose a detection method for complex disease causal SNP combinations from an optimal SNP dataset by using random forests with variable selection. Mapping the biological meanings of detected SNP combinations can help uncover complex disease mechanisms.

## Background

Detecting causal single nucleotide polymorphisms (SNPs) from genome-wide association studies (GWASs) has been focusing on measuring the statistical power of single SNPs, which have a relatively small effect on predicting disease susceptibility and ignore prior biological information about the target disease. Especially in complex diseases such as type 2 diabetes (T2D), the effect of each single SNP is too small to explain the disease association significantly.

To enhance the statistical power, we propose considering combinations of SNPs. Yang et al. discovered that estimates of variance explained by genome-wide SNPs are unbiased with the proportion of SNPs used to estimate genetic relationships in human height [1]. Although SNPs with relatively low statistical power are considered together, the statistical power is not significantly affected. In addition, Park et al. compared the discriminatory power of the risk models in Crohn's disease and prostate and colorectal (BPC) cancer and found that a risk model with all the predicted susceptibility loci has more discriminatory power than a risk model with only the known susceptibility loci [2]. Therefore, combinations of SNPs with not only significant SNPs that satisfy the genome-wide significance threshold but also common SNPs that have larger p-values than the genome-wide significance threshold may improve the prediction power of disease risk.

To rank SNPs and find SNP combinations, various methods are applied: Bayes factors [3], logistic regression [4,5], Hidden Markov Model (HMM) [6], Support Vector Machine (SVM), [7,8] and Random Forests (RF) [8-12]. Among the applied standard statistical methods and the machine learning-based methods, RF effectively ranks causal SNPs to detect SNP interactions [13,14].

Basically, RF is known to have a relatively low risk of overfitting compared to other machine learning algorithms [15]. However, if the number of variables is excessively larger than the number of samples, overfitting could occur. Furthermore, large datasets can increase the computational complexity greatly. Although Meanner et al. [9] and Wang et al. [10] did not apply specific threshold criteria for the GWAS dataset and applied 355,649 SNPs and 530,959 SNPs on RF analysis, respectively, previous causal SNP studies applied various threshold criteria to reduce the number of variables. Roshan et al. ranked T1D causal SNPs using RF and SVM from the Wellcome Trust Case Control Consortium (WTCCC) T1D dataset and the Genetics of Kidneys in Diabetes (GoKinD) T1D dataset by using Bonferroni thresholds [8]. Because of the computational capacity, Liu et al. selected the top 65,000 SNPs, which corresponded to a p-value threshold of 0.13 for SNP interaction screening, and selected 862 SNPs to analyze with RF [11]. To accommodate the computational requirements of SNPInterForest, Yoshida et al. selected the top 10,000 SNPs from a single SNP association analysis [12]. The optimal filtration method is required to avoid overfitting and to reduce the computational complexity.

From T2D GWA studies, approximately 40 causal individual SNPs have been identified [16]. However, the heritability of T2D is not yet fully understood and only about 10% of the T2D risk is explained by the causal SNPs that have been detected so far [17]. In addition, the accuracy of the T2D risk prediction with GWAS datasets from recent studies was approximately around 0.55-0.63, which is lower than that of other complex diseases such as T1D, Crohn's disease, rheumatoid arthritis, Alzheimer's disease, and multiple sclerosis [18]. The statistical simulation with heritability indicated that the accuracy of the T2D risk prediction can be improved to 0.8-0.9 if more common SNPs are combined [17]. Therefore, finding the missing heritability by combining common SNPs is essential to discover the T2D mechanism and clinical applications.

To detect T2D causal SNP combinations, Ban et al. tried to find SNP combinations from a dataset containing 408 SNPs in 462 T2D cases and 456 controls using SVM. From p-values that were less than 0.6, a SNP combination with 14 SNPs was selected using a p-value-based filtering method, and the prediction accuracy was 0.653 [7]. Ban et al. successfully suggested the possibility of T2D causal SNP combinations, but the size of the dataset was small and the accuracy was not significantly improved.

Recently, we presented T2D causal SNP combinations from an optimal SNP dataset and find the biological meaning of the detected causal SNP combination [19]. Our previous method was one of the first T2D SNP combination-finding studies using RF with biological meaning detection, even though RF has advantages for detecting SNP combinations. To avoid overfitting and to reduce the computational complexity, our previous method applies linkage disequilibrium (LD) pruning and finds an optimal SNP dataset by comparing the error rates of the selected SNP combinations from Bonferroni thresholds and p-value thresholds. In addition to our previous research, we apply expanded GSEA on not only T2D causal SNP combinations but also genome-wide SNPs to find the T2D associated functional modules and we compare the prediction error rates of T2D causal SNP combinations from an optimal SNP dataset and from a functional module-based filtration.

## Methods

### Linkage disequilibrium pruning based filtration

T2D association of single SNPs is analyzed by measuring the statistical power of individual markers. The WTCCC [3] GWAS dataset contains 500,567 SNP markers from 1,999 T2D cases and 3,004 controls. Quality control (QC) is applied for single SNP association analysis. To identify and remove poor quality samples, per-individual QC is applied as a sample missing genotype rate of > 3%. To identify and remove the poor quality SNPs, per-marker QC is applied as a SNP missing genotype rate of > 1%, minor allele frequency (MAF) < 1%, and Hardy-Weinberg Equilibrium (HWE) p-value ≤ 10^-4. ^After QC, 409,656 SNPs remained. For single SNP association analysis, Cochran-Armitage trend test statistics is applied using PLINK 1.07 (http://pngu.mgh.harvard.edu/purcell/plink/) [20].

To reduce the number of variables, LD pruning is used on 409,656 SNPs after the single SNP association analysis. Reducing the number of variables has advantages for reducing the computational complexity and avoiding the possibility of overfitting. In addition, to map SNP combinations at the gene level and the functional module level, LD pruning is an effective solution to avoid overestimation of a specific gene or a functional module containing multiple SNPs. The most significant SNP is selected among the SNPs in LD with r^2 ^> 0.8 within 1 Mb. After LD pruning, 42,798 SNPs were selected for the SNP combination analysis.

### Finding SNP combinations from an optimal SNP dataset

To reduce the computational complexity, the proposed method finds an effective threshold of the significance of SNPs by comparing the error rates of the SNP combinations from the Bonferroni threshold, the p-value rank-based threshold and the p-value range-based threshold criteria. Based on the Bonferroni threshold criteria, r is defined as the number of SNPs within the corrected p-value threshold that is determined as 0.05 divided by the total number of SNPs. The Bonferroni thresholds r, 2r, 5r, and 10r are applied to select SNP datasets. Based on the p-value rank-based threshold criteria, 500 SNP sets that are generated from the SNPs of p-value ranks 1-500, which are calculated using a cumulative approach, are fully tested to find the patterns of error rates. Furthermore, the p-value range-based threshold criteria are applied with p-value cutoffs of 0.01, 0.05 and 0.1 to 1.0 based on a cumulative approach. The error rates of SNP combinations from the SNP datasets with various threshold criteria are compared to find the optimal SNP dataset.

The RF algorithm is a combinational classifier that contains multiple classification trees to aggregate them into one classifier. Each classification tree is generated from a bootstrapped sample set, and the Gini index is measured for splitting. RF is selected to find SNP combinations because of its effective performance in ranking the causal SNPs. In addition, RF can detect SNPs with small statistical power because separate models are automatically fit to subsets of data from early splits in the tree [14]. To find an SNP combination from a GWAS dataset, RF with a variable selection algorithm is applied [21]. The R package varSelRF can select very small sets of features such as SNPs or genes that retain high predictive accuracy [22].

For the optimal parameter settings of varSelRF for finding a SNP combination from the WTCCC T2D dataset, various values are applied to mtryFactor (the multiplication factor to decide the number of variables for the splitting), ntree (the number of trees for the first forest), ntreeIterat (the number of trees for all additional forests), and vars.drop.frac (the drop fraction of variables at each iteration). The error rates are not significantly affected by ntree and ntreIterat when ntree is changed from 500 to 10,000 and ntreeIterat is changed from 200 to 4,000. However, the error rates decrease as the values of vars.drop.frac decrease from 0.35 to 0.2, even though the computation time is greatly increased. Furthermore, mtryFactor values from 0 to 13 are tested, and the error rates are smaller than 0.12 for mtryFactor values between 0.75 and 2. Therefore, the default values of arguments from varSelRF are accepted for the SNP combination analysis: mtryFactor = 1, ntree = 5,000, ntreeIterat = 2,000, and vars.drop.frac = 0.2.

### Mapping the biological meanings of SNP combinations and functional module-based filtration

T2D genes are collected to find the biological meanings of SNP combinations by using gene level mapping. T2D genes are collected from public disease gene databases such as OMIM [23], KEGG [24], and GAD [25]. From DrugBank [26], KEGG Drug and PharmGKB Drug [27] databases, 36 T2D drug targets were collected.

To discover the biological meaning and disease mechanism of a SNP combination that consisted of SNPs from diverse genes, an expanded gene set enrichment analysis (GSEA) is applied to test the disease association of functionally related genes [28]. Expanded GSEA can help to find the biological processes and pathways of underlying complex diseases.

Various functional modules are collected for a better understanding of the biological functions of a SNP combination (Table 1). First, pathway functional modules are collected from KEGG [24], KEGG Modules, BioCarta, Reactome [29], NCI-Nature [30], PANTHER [31], UniPathway [32], and MetaCyc [33]. Moreover, transcription factor (TF)-target functional modules and miRNA-target functional modules from promoters and 3'-untranslated region (UTR) motifs are collected from MSigDB [34]. To collect protein complex functional modules, COFECO [35], which contains data from Reactome, CORUM [36], Gene Ontology (GO) [37] cellular component category, PINdb [38], and Mpact [39] is selected. The GO biological process category is also collected to find the biological processes of selected SNPs. Compared with recently published studies, the functional module data used in the proposed method are increased in terms of both the number of resources and the number of gene sets [28,40,41]. Whereas recent GSEAs used gene sets in the range of 200-1,000, primarily from KEGG, GO, MSigDB and BioCarta, collected gene set data for the expanded GSEA contained 3,663 functional modules from 11 resources. Expanded GSEA applied Fishers Exact test using the collected functional module data. Expanded GSEA is applied on both whole WTCCC T2D dataset and detected SNP combination to find the significantly T2D associated functional modules and biological meaning of the SNP combination. Significantly T2D associated functional modules from expanded GSEA are selected for functional module-based filtration. Functional module-based filtration selects SNPs within the same functional module and selected SNP sets are tested to measure the effect sizes of functional modules.

**Table 1:** Databases used for enrichment analysis of expanded functional module.

Database	Gene Set Category	Web address
BioCarta	Pathway	http://www.biocarta.com/genes/index.asp
COFECO	Complex	http://piech.kaist.ac.kr/cofeco/
GO	Function	http://www.geneontology.org/
KEGG	Pathway	http://www.genome.jp/kegg/pathway.html
KEGG Modules	Pathway	http://www.genome.jp/kegg/module.html
MetaCyc	Pathway	http://metacyc.org/
MSigDB	TF-target, miRNA-target	http://www.broadinstitute.org/gsea/msigdb/index.jsp
NCI-Nature	Pathway	http://pid.nci.nih.gov/
PANTHER	Pathway	http://www.pantherdb.org/pathway/
Reactome	Pathway	http://www.reactome.org/
UniPathway	Pathway	http://www.grenoble.prabi.fr/obiwarehouse/unipathway

### Measurement of prediction error rates from random forest analysis

We compare the prediction error rates of SNP combinations and SNP sets from an optimal SNP dataset and from a functional module-based filtration. To measure the prediction error rates from RF, the R package varSelRF is applied. The default argument values (mtryFactor = 1, ntree = 5,000, ntreeIterat = 2,000, and vars.drop.frac = 0.2) are accepted for analyzing the gene sets and SNP sets. In addition to top T2D-associated gene sets, various SNP sets are analyzed with RF to measure the significance of T2D-associated gene sets.

## Results

### SNP combinations from an optimal SNP dataset

The detection of T2D causal SNP combinations considering common SNPs with low statistical power in single SNP association analysis may perform better in disease risk predictions because common SNPs can explain critical effects if they interact with other SNPs as a SNP combination. An optimal SNP combination can be selected by comparing the error rates from RF analysis with variable selection. To avoid overfitting and to reduce the computational complexity, the proposed method detects an optimal SNP dataset by comparing the error rates of SNP combinations from Bonferroni thresholds and p-value thresholds.

First, Bonferroni thresholds are applied to 42,798 SNPs selected from the WTCCC T2D GWAS dataset. In Table 2, r is the number of SNPs within the Bonferroni correction with p-values. While the T1D analysis from Roshan et al. found that the Bonferroni threshold of 2r improved the ranks of causal variants and achieved higher power, T2D analysis from the proposed method shows that 10r has higher power than r, 2r, and 5r. T2D is known to be more complex disease than T1D, and the disease risk prediction rates of T2D from previous studies were lower than the disease risk prediction rates of T1D. From the error rates of SNP combinations with Bonferroni threshold-based cutoff criteria, we can infer that T2D has more causal SNPs than previous studies, which used 10-20 SNPs.

**Table 2:** Error rates of SNP combinations from GWAS dataset with Bonferroni threshold based cutoff criteria.

Bonferroni threshold	Number of SNPs	No. of SNPs in SNP combination	Error rate
r	82	82	0.117553
2r	164	67	0.116953
5r	410	69	0.114954
10r	820	88	0.114154

**Figure 1:**
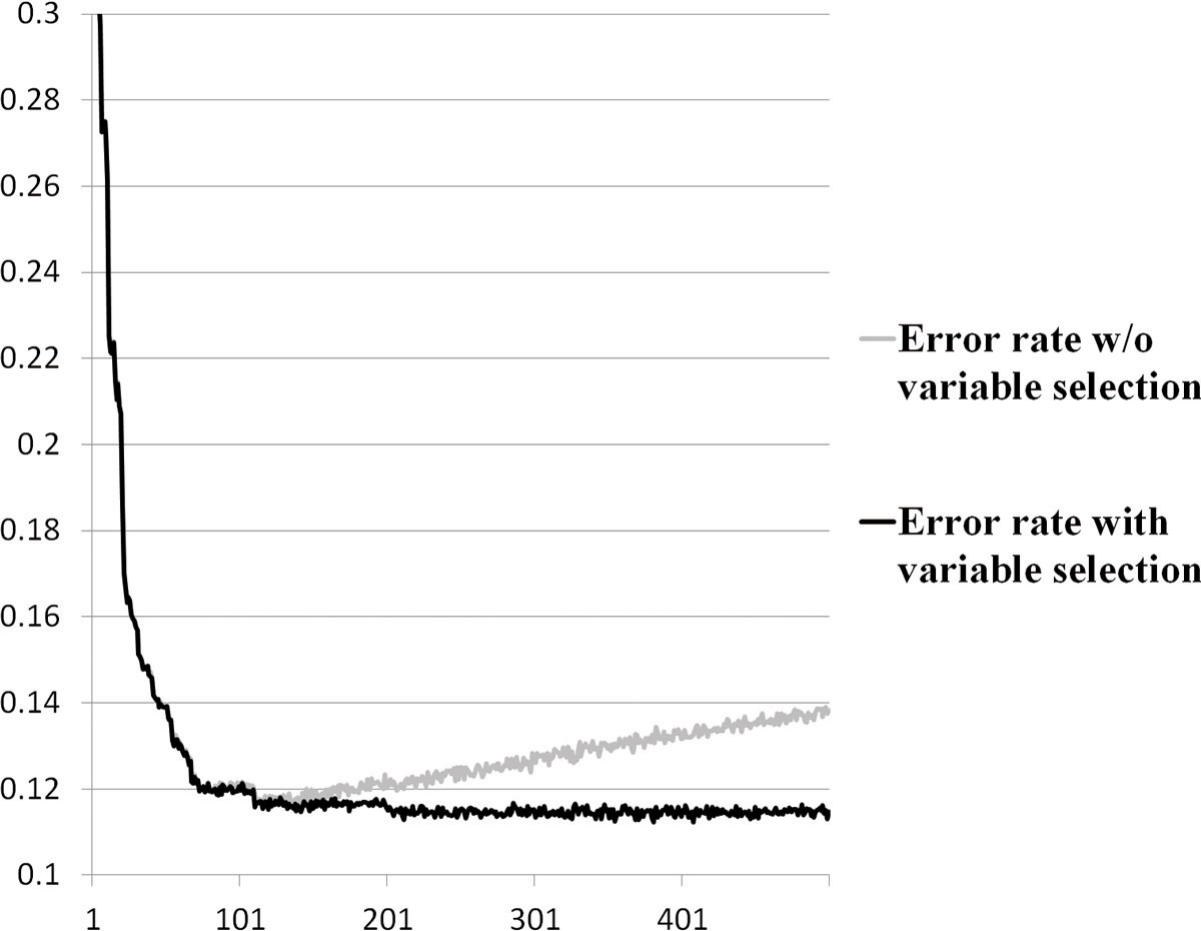
**Error rates of RF analysis with/without variable selection**.

Figure 1 shows the changes in error rates of RF analysis considering the top 500 ranked SNPs with or without variable selection. The error rate without variable selection is 0.3392 for the top-ranked SNP, and it dramatically decreases to 0.1171 with top 116 SNPs, which is almost same as the error rate that was calculated with variable selection with top 116 SNPs. However, the error rate without variable selection increases when the number of considered SNPs is increased to more than 116 SNPs, while the error rate with variable selection is stable. RF with variable selection effectively selects T2D causal SNP combinations consisting of 1-161 SNPs (average 76.55 SNPs) from 1-500 SNPs, and has low error rates. As shown in Figure 1, the number of T2D causal SNPs for the T2D disease risk prediction could be more than that predicted by previous studies, which used 10-20 diabetes-related SNPs [7,17]. The proposed method shows that SNP combinations of more than 100 SNPs have lower error rates than SNP combinations of the top 10-20 SNPs. Furthermore, even though the error rates with variable selection seem almost stable with 116 SNPs, the error rate is slightly decreased when the number of considered SNPs is increased. Therefore, we expanded the threshold criteria to include p-values from 0.01 to 1.0.

As shown in Table 3, the error rates of a SNP combination is measured with p-value range-based cutoff criteria, with p-value cutoffs of 0.01, 0.05, and 0.1-1.0. Although p-value ranges smaller than 0.05 are usually accepted as a standard cutoff, most of the error rates of SNP combinations from p-value ranges larger than 0.05 were lower than those from p-value ranges smaller than 0.05. From Table 2 and Table 3, we can infer that if even a SNP was without significant p-value from a single SNP association analysis, the prediction power of a SNP combination could be boosted by including a SNP with low statistical power. Error rates tend to decrease from p-value ranges less than 0.01 to p-value ranges less than 0.6 when more SNPs with low statistical power are considered together. The best error rate of SNP combinations was 0.102538 with 101 SNPs from the 26,743 SNPs that had a p-value less than 0.6. Therefore, SNP combination with 101 SNPs from a p-value range less than 0.6 is selected for the biological meaning mapping.

**Table 3:** Error rates of SNP combinations from a GWAS dataset with p-value range-based cutoff criteria.

p-value range	No. of SNPs in p-value range	No. of selected SNPs in SNP combination	Error rate
<0.01	854	114	0.116953
<0.05	2960	83	0.114754
<0.1	5297	95	0.115131
<0.2	9831	91	0.114731
<0.3	14192	104	0.102938
<0.4	18407	87	0.104937
<0.5	22612	134	0.106136
<0.6	26743	101	0.102538
<0.7	30797	59	0.109934
<0.8	34789	85	0.104138
<0.9	38815	60	0.113332
<1	42798	83	0.114731

**Figure 2:**
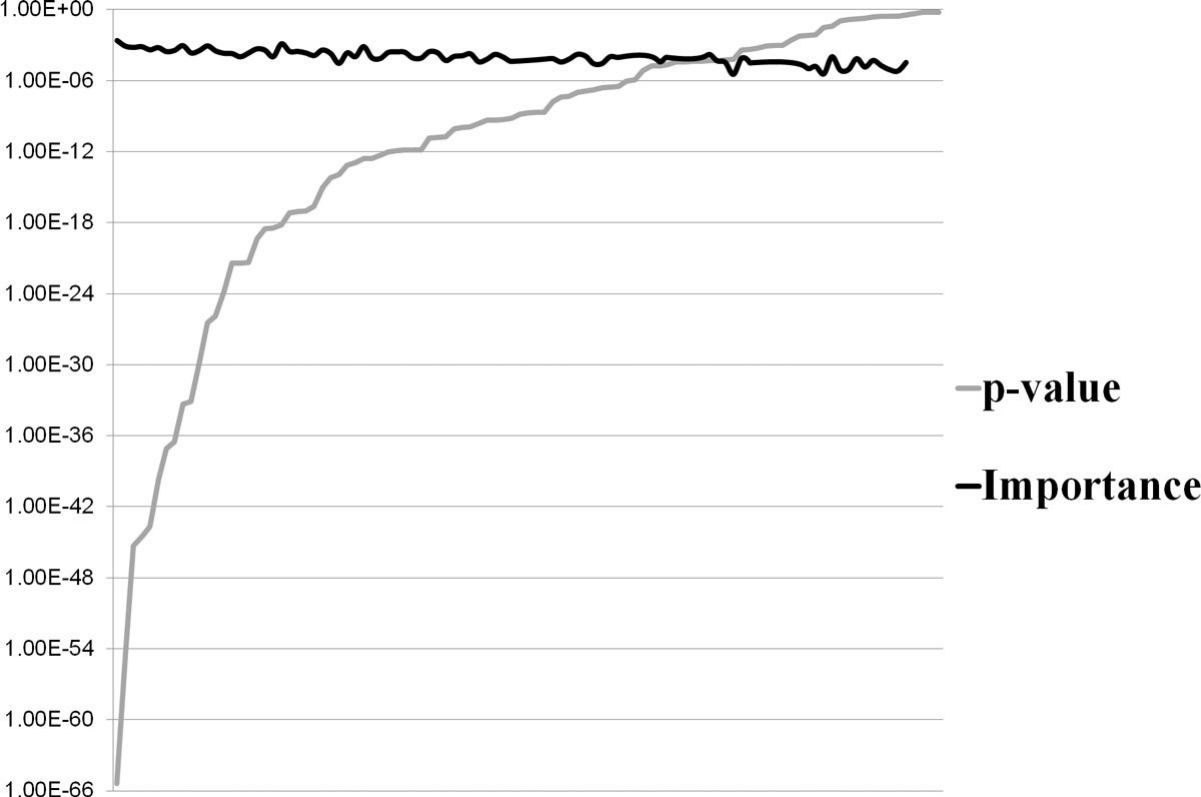
**P-values and variable importance values of SNPs from the SNP combination**.

Figure 2 indicates the relationship between the p-values and the variable importance values of 101 SNPs from RF analysis. Although the p-values of SNPs from the SNP combination are dramatically increased, the variable importance values maintained their stability. From 101 SNPs in the SNP combination, 13 SNPs have a p-value that is greater than 0.05. Although the p-values of 13 SNPs are much higher than those of other selected SNPs in the SNP combination, the variable importance of the 13 SNPs calculated from RF analysis have similar value to those of other SNPs; for most SNPs, lower p-values results in higher importance values. In addition, compared with SNPs that are not selected in the SNP combination, the selected SNPs have significant variable importance values within a small range. The error rates of SNP combinations tend to decrease when the p-value range is increased (Table 3) because SNPs with high p-values and high importance values may play important roles through interactions with other SNPs and genes.

### Biological meaning mappings of SNP combinations

The selected SNP combination from RF analysis contains 101 SNPs that can be mapped to 107 nearby genes (see Additional file 1 for the Additional Table 1). To find the biological meaning of the selected SNP combination, multiple levels of information are matched. SOS1 and FTO are found to be T2D-related genes by matching with collected disease genes. In addition, TFB1M is recently revealed as a T2D-related gene with a common variant that is associated with insulin secretion. [42]

From the collected 5,289 functional modules, 1,305 functional modules are selected with 101 SNPs from RF analysis. Among the 1,305 functional modules, 87 functional modules recorded a false discovery rate (FDR) less than 0.05 with Fisher's exact test. Table 4 shows the pathway functional modules matched with 101 SNPs from the SNP combination. The epidermal growth factor (EGF) receptor (ErbB1) signaling pathway is known to affect T2D by regulating pancreatic fibrosis [43]. The platelet-derived growth factor (PDGF) signaling pathway, which controls islet regeneration and proliferation, is inactivated in T2D cases [44]. In addition, the Rho GTPase pathway is activated in the T2D model and in cell lines with high concentrations of glucose [45].

**Table 4:** Pathway functional modules from SNP combination.

Functional Module Name	Category	# of genes	# of genes in functional module	p-value	FDR
Focal adhesion	KEGG	6	175	3.144E-03	7.982E-03
Hemostasis	Reactome	6	207	7.080E-03	1.091E-02
ErbB1 downstream signaling	NCI-Nature	4	101	9.465E-03	1.662E-02
Endometrial cancer	KEGG	3	48	7.073E-03	1.755E-02
Formation of Platelet plug	Reactome	4	106	1.116E-02	1.840E-02
PDGF signaling pathway	PANTHER	4	108	1.190E-02	2.053E-02
Viral myocarditis	KEGG	3	54	9.792E-03	2.506E-02
BCR signaling pathway	NCI-Nature	3	63	1.487E-02	2.703E-02
amine and polyamine degradation	UniPathway	1	4	3.236E-02	3.236E-02
Rho GTPase cycle	Reactome	3	73	2.196E-02	3.420E-02
Regulation of RAC1 activity	NCI-Nature	2	26	1.900E-02	3.498E-02
Signaling by Rho GTPases	Reactome	3	73	2.196E-02	3.633E-02
Thrombin-mediated activation of PARs	Reactome	1	3	2.437E-02	3.841E-02
Fc epsilon RI signaling pathway	KEGGM	3	63	1.487E-02	4.051E-02
EGF receptor (ErbB1) signaling pathway	NCI-Nature	4	132	2.313E-02	4.096E-02
					

Table 5 demonstrates a part of the significant TF-target functional modules and miRNA-target functional modules from the SNP combination. Among the TFs from the significant TF-target functional modules, signal transducer and activator of transcription 4 (STAT4), androgen receptor (AR), pre-B-cell leukemia transcription factor 1 (PBX1), and paired box 6 (PAX6) are matched with T2D-related genes. In addition, signal transducer and activator of transcription 5 (STAT5) activity in pancreatic β-cells showed susceptibility to T2D [46]. Organic cation transporter 1 (OCT1) is related to the hepatic uptake of metformin, which is one of the most used drugs for T2D, and the genetic variation in the OCT1 gene can affect individual drug response to metformin [47]. From GO biological process category, angiogenesis is the most significant functional module, with an FDR value of 3.15E-02, and suppressed angiogenesis is one of the characteristic features of T2D complication [48].

**Table 5:** TF-target functional modules and miRNA-target functional modules from SNP combinations.

Functional Module Name	Category	# of genes	# of genes in functional module	p-value	FDR
TGACAGNY_V$MEIS1_01	TF-target	15	620	1.553E-04	1.901E-04
V$STAT4_01	TF-target	8	200	2.348E-04	2.786E-04
YTATTTTNR_V$MEF2_02	TF-target	13	545	5.034E-04	6.157E-04
V$CDP_02	TF-target	5	89	8.079E-04	9.780E-04
V$RORA1_01	TF-target	7	193	1.037E-03	1.249E-03
V$YY1_01	TF-target	7	195	1.101E-03	1.262E-03
TGCCAAR_V$NF1_Q6	TF-target	12	545	1.658E-03	1.731E-03
TTGTTT_V$FOXO4_01	TF-target	24	1549	1.435E-03	1.873E-03
V$OCT1_06	TF-target	7	218	2.086E-03	2.121E-03
V$NKX25_01	TF-target	5	102	1.491E-03	2.128E-03
V$CDC5_01	TF-target	7	217	2.032E-03	2.720E-03
V$AR_Q6	TF-target	6	178	3.419E-03	3.618E-03
V$POU6F1_01	TF-target	6	185	4.129E-03	4.403E-03
V$POU3F2_01	TF-target	4	83	4.764E-03	5.687E-03
V$STAT5A_03	TF-target	6	198	5.732E-03	5.794E-03
V$STAT6_02	TF-target	6	192	4.944E-03	5.855E-03
V$EVI1_03	TF-target	3	44	5.546E-03	7.165E-03
V$PBX1_01	TF-target	6	194	5.197E-03	7.461E-03
V$SRY_02	TF-target	6	196	5.460E-03	7.484E-03
V$AFP1_Q6	TF-target	6	204	6.607E-03	7.991E-03
ACTTTAT,MIR-142-5P	miRNA-target	7	254	4.854E-03	9.791E-03

### Expanded gene set enrichment analysis of WTCCC T2D dataset

From the 42,798 SNPs filtered from the WTCCC T2D GWAS dataset, 451 T2D-associated gene sets with a p-value threshold of <0.05 are detected from the expanded GSEA to match with the collected T2D-associated genes. In all, 2,112 gene sets contains T2D-associated genes from 3,663 expanded gene sets. Surprisingly, 441 gene sets contains T2D-associated genes from the detected 451 T2D-associated gene sets from the expanded GSEA. Moreover, the average number of T2D-associated genes from the selected 441 gene sets with expanded GSEA is 11.188, whereas the average number of T2D-associated genes from selected 2,121 gene sets from total expanded gene sets is 6.554.

The proposed expanded GSEA successfully selected T2D-related gene sets with superior performance than previous analyses regarding significance and coverage. Table 6 displays all selected pathways from expanded GSEA with a p-value < 0.05 in the WTCCC T2D dataset. In total, 34 pathway functional modules were selected with expanded GSEA, while 9 pathways were selected from Perry et al.'s analysis with a p-value < 0.05 [49]. Compared with selected pathways by Perry et al., the WNT signaling pathway was the most significant pathway gene set in both analyses. The WNT signaling contains TCF7L2 which is significantly associated with T2D in GWAS and may influence to T2D by affecting GLP-1 levels [50,51]. Compared with functional modules from SNP combination and functional modules from genome-wide SNPs, focal adhesion, EGFR (ErbB1) signaling pathway, and hemostasis functional modules are enriched in both expanded GSEAs.

**Table 6:** Selected pathways from expanded GSEA with a p-value < 0.05 in the WTCCC T2D dataset

Database	Pathway	Size	p-value	FDR
PANTHER	WNT signaling pathway	130	0.001	0.056
KEGG	Calcium signaling pathway	105	0.002	0.073
KEGG	Pathways in cancer	193	0.002	0.081
KEGG	Focal adhesion	132	0.004	0.090
KEGG	Neuroactive ligand-receptor interaction	127	0.01	0.153
KEGG	MAPK signaling pathway	146	0.012	0.168
KEGG	Cell adhesion molecules (CAMs)	61	0.016	0.208
NCI-Nature	Regulation of RhoA activity	73	0.03	0.287
PANTHER	Huntington disease	58	0.03	0.277
Reactome	Signalling by NGF	107	0.03	0.359
KEGG	Non-small cell lung cancer	38	0.031	0.303
KEGG	Natural killer cell mediated cytotoxicity	56	0.035	0.232
PANTHER	Muscarinic acetylcholine receptor 1 and 3 signaling pathway	30	0.035	0.325
Reactome	Integrin cell surface interactions	57	0.036	0.330
KEGG	Axon guidance	74	0.036	0.419
Reactome	Cell Cycle, Mitotic	120	0.039	0.254
NCI-Nature	Notch signaling pathway	49	0.04	0.259
NCI-Nature	Signaling events mediated by focal adhesion kinase	42	0.041	0.260
NCI-Nature	EGF receptor (ErbB1) signaling pathway	94	0.043	0.379
Reactome	Gene Expression	146	0.043	0.379
KEGG	ErbB signaling pathway	55	0.043	0.379
NCI-Nature	Neurotrophic factor-mediated Trk receptor signaling	67	0.044	0.384
PANTHER	Beta1 adrenergic receptor signaling pathway	24	0.044	0.379
NCI-Nature	Hypoxic and oxygen homeostasis regulation of HIF-1-alpha	46	0.044	0.274
PANTHER	Heterotrimeric G-protein signaling pathway-Gq alpha and Go alpha mediated pathway	68	0.045	0.492
KEGG	Cytokine-cytokine receptor interaction	105	0.046	0.393
Reactome	Signaling in Immune system	118	0.046	0.399
Reactome	G-protein mediated events	27	0.047	0.346
NCI-Nature	Thromboxane A2 receptor signaling	40	0.047	0.346
PANTHER	FGF signaling pathway	60	0.048	0.403
KEGG	Adherens junction	52	0.048	0.406
KEGG	Leukocyte transendothelial migration	62	0.049	0.356
Reactome	Integration of energy metabolism	79	0.049	0.363
Reactome	Hemostasis	145	0.049	0.526

### Measurement of prediction error rates from random forest analysis

To measure the susceptibility of functional modules, we measured prediction error rates of functional modules from RF analysis. Table 7 presents the RF-based prediction error rates of SNP sets from functional module-based filtration and SNP combinations with various thresholds from the WTCCC T2D dataset. From the 42,798 SNPs filtered from WTCCC T2D GWAS dataset, 66 T2D-associated functional modules with FDR < 0.05 were selected from the expanded GSEA and all 66 T2D-associated functional modules were analyzed with RF. Among these 66 functional modules, the lowest error rate was 32.12%, and the average prediction error rate from 66 functional modules was 36.98%. For the comparison, the average prediction error rate of 66 SNP sets consisting of randomly selected 1615 SNPs was measured as 36.57%, which contained nearly identical numbers as the average number of SNPs in 66 functional modules. Compared to randomly selected SNP sets, functional modules showed no significance on T2D.

**Table 7:** RF-based prediction error rates of SNP sets from functional module-based filtration and SNP combinations with various thresholds from the WTCCC T2D dataset.

Dataset	Functional Module Description	Number of SNPs	Error Rate	Number of Selected SNPs	Error Rate with Variable Selection
MIR	CACTGCC,MIR-34A, MIR-34C,MIR-449	1876	39.02%	6	32.12%
TF	V$NKX25_02	1678	38.56%	59	33.06%
MIR	ACTTTAT,MIR-142-5P	1572	38.32%	35	33.42%
Average	Average of results from 66 functional modules	1614.86	39.18%	32.24	36.98%
Random Average	Average of results from 66 SNP sets consisting of randomly selected 1615 SNPs	1615	38.77%	113.17	36.57%
Combination	Top 5 functional modules	7590	38.96%	36	32.78%
Combination	Top 66 functional modules	25663	38.66%	62	17.43%
p-value based	Top 1 SNP	1	33.93%	1	33.93%
p-value based	Top 10 SNPs	10	27.19%	10	27.19%
p-value based	SNPs with Bonferroni threshold	82	11.76%	82	11.76%
p-value based	SNPs with p-value < 0.01	854	14.79%	114	11.70%
p-value based	All SNPs	42798	37.02%	83	11.47%

Various p-value based SNP sets were also applied as references. The prediction error rate of top 1 SNPs of p-value rank was 33.93%, and the prediction error rate of top 10 SNPs was 27.19%, which are relatively lower than the functional module based prediction error rates when considering 1500-2000 SNPs. On the basis of the Bonferroni threshold criteria, a SNP set with top 82 SNPs within the Bonferroni correction with p-values was selected, and the prediction error rate was 11.76%. The prediction error rate of a SNP set which was measured with a p-value cutoff of 0.1 was 11.70% and the prediction error rate of a SNP set with whole 42798 SNPs was 11.47%. Compared with various p-value-based SNP sets, no specific functional module (pathway, GO function, TF-target, miRNA-target, and protein complex) could significantly explain the T2D association alone.

To enhance the predictive power, the combination of top 5 functional modules and top 66 functional modules was applied. However, the prediction error rates were slightly reduced, although the number of considered SNPs was dramatically increased.

## Discussion

Various thresholds, including Bonferroni correction thresholds and p-value-based thresholds, are tested to find the optimal threshold with considering SNPs with low statistical power. SNP combinations that contain SNPs with low statistical power had lower error rates than SNP combinations with only significant SNPs. With the consideration of common SNPs with low statistical power, the disease risk prediction rate can be improved, especially for complex diseases.

Notable disease genes could be found from SNP combinations. From the selected SNP combination, there are still many genes that are not yet identified as a T2D-related disease gene. SNP combinations have high statistical power. In addition, the activation or inhibition of a T2D-related pathway could prevent and cure T2D and T2D complications. For example, the Rho GTPase pathway inhibitor can prevent T2D development [43].

No specific functional modules from the T2D-associated gene sets shows significance in T2D development from the RF-based prediction error rates. From the results of the measurement of prediction error rates from RF analysis, we can infer that significant T2D SNPs and genes with high importance are widespread in the genome and are not concentrated in a specific functional module. The expansion of functional modules with protein-protein interaction network may increase the susceptibility of T2D.

## Conclusions

To overcome the low statistical power of single SNPs, considering multiple SNPs together becomes a solution for analyzing complex diseases. A T2D causal SNP combination is detected using RF with variable selection from an optimal SNP dataset filtered with a p-value threshold and LD pruning. From the WTCCC T2D GWAS dataset, 101 SNPs are selected with a SNP combination. Not only significant SNPs but also common SNPs with low statistical power are combined as a SNP combination. Mapping the SNP combination at the SNP, gene, and functional module levels gives clues to the relationship with T2D. Functional module-based filtration is also tested using T2D associated functional modules from genome-wide SNPs and the results showed no significance compared to randomly selected SNP sets. The proposed method can detect a SNP combination with considering SNPs with low statistical power. Additionally this method can reveal the biological meaning of the detected SNP combination by mapping functional modules and mapping the T2D-related information at multiple levels including disease genes.

## Competing interests

The authors declare that they have no competing interests.

## Authors' contributions

CK designed and implemented the proposed method and wrote the manuscript. HY participated in the implementation of the proposed method. GSY designed and directed this study, and reviewed the manuscript. All authors worked on and approved the final manuscript.

## Supplementary Material

Additional file 1 Selected SNPs from the type 2 diabetes causal SNP combination Click here for file

## Declarations

The publication costs for this article were partially funded by the Basic Science Research Program through the National Research Foundation of Korea (NRF) grant 2012R1A1A2008510 and the NRF grant No. 2012-0001001 funded by the Ministry of Education, Science and Technology (MEST) of Korean government.

This article has been published as part of *BMC Medical Informatics and Decision Making *Volume 13 Supplement 1, 2013: Proceedings of the ACM Sixth International Workshop on Data and Text Mining in Biomedical Informatics (DTMBio 2012). The full contents of the supplement are available online at http://www.biomedcentral.com/bmcmedinformdecismak/supplements/13/S1.
