# Supplementary material for: Finding type 2 diabetes causal single nucleotide polymorphism combinations and functional modules from genome-wide association data
Source: BMC Med Inform Decis Mak. 2013 Apr 5;13(Suppl 1):S3. doi: 10.1186/1472-6947-13-S1-S3 (PMC3618247; doi:10.1186/1472-6947-13-S1-S3)
Supplement: Additional file 1 — Selected SNPs from the type 2 diabetes causal SNP combination [file 1472-6947-13-S1-S3-S1.PDF]

Additional file 1

Selected SNPs from the type 2 diabetes causal SNP combination

SNP	Chr.	Position (bp)	Nearby gene	Region
6763532	3	160701816	PPM1L	intron
10494526	1	180279710	ACBD6	intron
17116117	11	113801591	HTR3B	intron
10184046	2	39336703	SOS1	intron
12303046	12	86723435	MGAT4C	intron
41412947	2	204594247	CD28	intron
41510648	18	50419556	DCC	intron
41470451	18	28608758	DSC3	intron
41506144	10	13876687	FRMD4A	intron
1805625	3	180691661	FXR1	intron
16876548	5	78425046	BHMT	intron
41368550	16	80694253	CDYL2	intron
1801741	3	15296604	SH3BP5	3'UTR
9638885	7	33144659	RP9	intron
41486250	3	170608213	EIF5A2	3'UTR
41501252	2	216192167	ATIC	intron
41535452	10	101124600	CNNM1	intron
41334651	6	155613012	TFB1M	intron
41381548	5	147941428	HTR4	intron
3743350	15	67677239	IQCH	intron
3777582	6	45914028	CLIC5	intron
41515446	2	42808856	MTA3	intron
28603187	22	36398593	RBFOX2	intron
7292491	22	26195440	MYO18B	intron
41385046	1	192544672	RGS1	nearGene-5
41449646	16	50735455	NOD2	intron
41515544	14	69364030	ACTN1	intron
12064327	1	201384778	TNNI1	intron
41529047	1	185040789	RNF2	intron
16848754	1	201455883	CSRP1	intron
41450546	20	44574459	PCIF1	cds-synon

3780786	9	136816065	VAV2	intron
41355251	14	33785704	NPAS3	intron
41364849	2	25789594	DTNB	intron
41411251	4	36103966	ARAP2	intron
10170191	2	142526005	LRP1B	intron
12325390	16	15843441	MYH11	intron
41510050	17	64482038	PRKCA	intron
41359646	8	32539521	NRG1	intron
41434950	8	51244654	SNTG1	intron
41511849	21	18930386	CXADR	intron
41389844	4	101386945	EMCN	intron
9478918	6	151358299	MTHFD1L	intron
285584	12	41946539	PDZRN4	missense
41479544	18	55472316	ATP8B1	nearGene-5
10996819	10	67759006	CTNNA3	intron
41438745	7	102561170	FBXL13, LRRC17	intron
41363348	16	6725968	RBFOX1	intron
862028	14	74996603	LTBP2	intron
41482053	5	9301803	SEMA5A	intron
883816	9	129873632	ANGPTL2, RALGPS1	intron
3742781	14	75472502	EIF2B2	intron
7666328	4	115783305	NDST4	intron
41464948	11	75327712	MAP6	intron
41436848	5	179177201	MAML1	intron
41448248	3	149675245	RNF13	intron
2243880	7	18659363	HDAC9	intron
41386051	3	99798892	FILIP1L, C3orf26	intron
1525791	7	39156558	POU6F2	intron
168841	16	8958463	CARHSP1	intron
41487148	6	90326657	ANKRD6	intron
2069698	5	75916887	F2RL2, IQGAP2	intron
41526651	1	9404693	SPSB1	intron
9926289	16	53820503	FTO	intron

41482148	3	42151018	TRAK1	intron
41434052	12	107906294	BTBD11	intron
968582	21	41278955	PCP4	intron
41456047	14	65184505	PLEKHG3	intron
9287594	2	236890752	AGAP1	intron
17461148	4	44414209	KCTD8	intron
2145028	14	35307031	BAZ1A	intron
1054469	7	106959913	COG5	intron
13126272	4	185731940	ACSL1	intron
1048327	15	101609737	LRRK1	3'UTR
7569522	2	161346447	RBMS1	intron
41422344	18	52945557	TCF4	intron
41401648	3	151155383	IGSF10	cds-synon
12514672	5	35188257	PRLR	intron
2347624	6	71500873	SMAP1	intron
1957779	14	63669647	RHOJ	nearGene-5
41427844	11	128637259	FLI1	intron
41397146	1	34400204	CSMD2	intron
41496451	11	95635241	MTMR2	intron
41375748	1	163114057	RGS5	3'UTR
1800753	11	6636668	ILK, TPP1, TAF10	intron
41497550	3	169168595	MECOM	intron
41345951	1	208231435	PLXNA2	intron
4309965	5	112377617	MCC	intron
11102353	1	112426519	KCND3	intron
4773187	13	111111455	COL4A2	intron
41408148	15	32404959	CHRNA7	intron
41471245	12	109019243	SELPLG	intron
6441345	3	160696867	PPM1L	intron
41472346	2	158948074	UPP2	intron
41525050	1	201268263	PKP1	intron
4742259	9	6755968	KDM4C	nearGene-5
41449048	2	153028147	STAM2	intron
41514650	3	125697081	ROPN1B	intron
41499252	7	28338099	CREB5	nearGene-5

41409044	10	84425889	NRG3	intron
41500451	4	109034946	LEF1	intron
